# Older adults and sexual well-being: A qualitative study in Portugal and Slovenia

**DOI:** 10.1192/j.eurpsy.2022.372

**Published:** 2022-09-01

**Authors:** S. Von Humboldt, J.A. Ribeiro-Gonçalves, A. Costa, G. Low, E. Benko, I. Leal

**Affiliations:** 1 ISPA – Instituto Universitário, Lisbon, Portugal, William James Research Center, Lisbon, Portugal; 2 Instituto Universitário, Ispa, Lisbon, Portugal; 3 University of Alberta, Faculty Of Nursing, Edmonton, Canada; 4 University of Primorska, Faculty Of Health Sciences, Izola, Slovenia

**Keywords:** Qualitative study, Sexual well-being, Cross-cultural, Older Adults

## Abstract

**Introduction:**

Beyond living longer, it is increasingly important to live with more and better health during aging (1). Sexual well-being (SWB) was found to contribute to health and well-being in old age and is highly under-researched in the older population (2).

**Objectives:**

This study aims to analyze SWB in a cross-cultural way through older Portuguese and Slovenian older samples.

**Methods:**

We interviewed 136 older participants with an average age of 71.6 years old. Participants were Portuguese and Slovenian and lived in the community. Participants were subjected to semi-structured interviews and these were subjected to a content analysis process.

**Results:**

The content analysis indicated nine themes related to SWB: self-reported good health; demonstrations of love; non-sexual joint activities; overall well-being and quality of life; partner support; positive self-image; being independent and active; sexual compatibility; and masturbation. Portuguese older adults experience their SWB associated mainly with self-reported good health and demonstrations of love, while Slovenians older adults associate their SWB mainly with non-sexual joint activities and overall well-being and quality of life.

**Conclusions:**

The themes found in this study are fundamental evidence for cultural interventions and guidelines outlining in the context of sexual health in aging, mainly due to the scarcity of knowledge of SWB among older adults. 1.von Humboldt S et al. Sexual expression in old age: How older adults from different cultures express sexually? Sex Res Social Policy. 2020;1-15. 2.von Humboldt S et al. Are older adults satisfied with their sexuality? Outcomes from a cross-cultural study. Educ Gerontol. 2020;46:284-293.

**Disclosure:**

No significant relationships.

